# Transcriptome Analysis Revealed Potential Mechanisms of Resistance to *Trichomoniasis gallinae* Infection in Pigeon (*Columba livia*)

**DOI:** 10.3389/fvets.2021.672270

**Published:** 2021-09-14

**Authors:** Jingwei Yuan, Aixin Ni, Yunlei Li, Shixiong Bian, Yunjie Liu, Panlin Wang, Lei Shi, Adamu Mani Isa, Pingzhuang Ge, Yanyan Sun, Hui Ma, Jilan Chen

**Affiliations:** ^1^Institute of Animal Science, China Academy of Agricultural Science, Beijing, China; ^2^Department of Animal Science, Usmanu Danfodiyo University, Sokoto, Nigeria

**Keywords:** pigeon, *Trichomoniasis gallinae* resistance, long non-coding RNA, mRNA, differential expression analysis

## Abstract

*Trichomoniasis gallinae* (*T. gallinae*) is one of the most pathogenic parasites in pigeon, particularly in squabs. Oral cavity is the main site for the host-parasite interaction. Herein, we used RNA-sequencing technology to characterize lncRNA and mRNA profiles and compared transcriptomic dynamics of squabs, including four susceptible birds (S) from infected group, four tolerant birds (T) without parasites after *T. gallinae* infection, and three birds from uninfected group (N), to understand molecular mechanisms underlying host resistance to this parasite. We identified 29,809 putative lncRNAs and characterized their genomic features subsequently. Differentially expressed (DE) genes, DE-lncRNAs and cis/trans target genes of DE-lncRNAs were further compared among the three groups. The KEGG analysis indicated that specific intergroup DEGs were involved in carbon metabolism (S vs. T), metabolic pathways (N vs. T) and focal adhesion pathway (N vs. S), respectively. Whereas, the cis/trans genes of DE-lncRNAs were enriched in cytokine-cytokine receptor interaction, toll-like receptor signaling pathway, p53 signaling pathway and insulin signaling pathway, which play crucial roles in immune system of the host animal. This suggests *T. gallinae* invasion in pigeon mouth may modulate lncRNAs expression and their target genes. Moreover, co-expression analysis identified crucial lncRNA-mRNA interaction networks. Several DE-lncRNAs including MSTRG.82272.3, MSTRG.114849.42, MSTRG.39405.36, MSTRG.3338.5, and MSTRG.105872.2 targeted methylation and immune-related genes, such as JCHAIN, IL18BP, ANGPT1, TMRT10C, SAMD9L, and SOCS3. This implied that DE-lncRNAs exert critical influence on *T. gallinae* infections. The quantitative exploration of host transcriptome changes induced by *T. gallinae* infection broaden both transcriptomic and epigenetic insights into *T. gallinae* resistance and its pathological mechanism.

## Introduction

Pigeons (*Columba livia*) have been raised for sport (racing breeds), exhibition (fancy breeds), food (meat-type breeds), and scientific research by humans for a long time. Pigeon parasitic and other pathogenic infections are prevalent in several countries, becoming an issue where pigeons are of great importance (1). *Trichomoniasis gallinae* (*T. gallinae*) is a kind of trichomoniasis extremely common in domestic pigeons. Generally, pigeons suffering from trichomoniasis shed the parasites through the saliva and the crop milk, leading to severe lesions or clinical signs in nestling and young pigeons. *T. gallinae* pose a major health and economic burden to the pigeon industry (2). In practice, birds showed considerable variation in resistance to the parasite, therefore, understanding the mechanism of resistance to *T. gallinae* and breeding pigeons for enhanced resistance would provide a sustainable long-term solution for reducing the burden poses by *T. gallinae* infections.

Long non-coding RNA (lncRNA) is a class of RNA transcripts longer than 200 nucleotides with coding potential. LncRNAs and mRNAs share many common features, such as 5′ 7-methylguanosine cap and a 3′ poly(A) tail (3). Depending on localization and specific interactions with DNA, RNA and proteins, lncRNAs regulate gene expression at various levels through diverse mechanisms, including mediating protein localization, interacting with chromatin modification complexes and affecting rate of transcription (4). LncRNAs are also involved in posttranscriptional processes, including alternative splicing, mRNA cleaving and decaying, protein translation and stability (5). The widespread application of transcriptome sequencing affirmed that lncRNAs play a crucial role in the development and activation of immune cells during different parasitic infections (6). During chronic whipworm infection, host upregulated numerous genes related to the immune system including interferons, immunoglobulins and tumor necrosis factors to limit damage (7). In *Plasmodium falciparum* infections, the joint transcriptomes of human and parasite revealed that genes of the innate immune response pathway including TLR2 and TICAM2 are correlated with the severity of the malarial infection (8). After *T. gondii* infection, lncRNAs impaired the secretion of some cytokines such as IL-12, TNF-α, IL-1β, and IFN-γ by downregulating UNC93B1 expression in human macrophage cells (9, 10).

However, transcriptome studies in domestic pigeon resistance to parasite infections are very limited, especially for the non-coding RNAs. Several studies in other animals reported that lncRNAs and circRNAs exert critical influence on hosts resistance to *Eimeria necatrix* (11). Furthermore, it is obvious that the transcriptomes of pigeon are inadequately characterized compared to other livestock species. Thus, the discovery and functional annotation of lncRNAs in pigeons is necessary and valuable. Previous studies have revealed that lncRNAs play important roles in regulating sperm mobility and lactation in pigeons (12, 13). Research on the roles of lncRNAs and mRNAs played in resistance to parasitic infections in pigeon is therefore needed.

To advance our knowledge of host response to *T. gallinae* infection, the comprehensive transcriptomes of oral mucosa from 11 newly hatched pigeons with different resistance to *T. gallinae* infections were analyzed. On the basis of high-throughput transcriptomic data, the objectives of this study were to: (1) assess the expression profiles of lncRNA transcriptome in pigeon oral mucosa; (2) identify candidate genes and lncRNAs associated with *T. gallinae* infections and (3) conduct co-expression network analysis to identify interactions between differentially expressed genes and lncRNAs with regard to their underlying roles in resisting *T. gallinae* invasion. These would provide a basis understanding for the potential effects of genes and lncRNAs on pigeons resistance to *T. gallinae* infection.

## Materials and Methods

### *T. gallinae* Inoculation and Examination

A total of 135 days old White King pigeon squabs were randomly divided into two groups (treatment vs. control), and raised in two separate isolators under same conditions. Squabs of the treatment group (*n* = 100) were nasally inoculated with 0.5 mL culture containing 5 × 10^6^ parasites/mL of *T. gallinae* strain, meanwhile, control group (*n* = 35) was nasally administered with equivalent volume of culture. The inoculation was conducted once a day for the first 3 days of the squabs. At 1, 2, 3, 4, and 7 days post inoculation (dpi) of the first inoculation, oral swabs were collected from each bird and placed in 1.5 mL sterile tubes containing stroke-physiological saline solution, at pH 6.8 and 37°C. The numbers of parasites were observed by microscope and counted on hemocytometer. At 3 dpi, squabs from the infected group were dichotomized into susceptible and tolerant group, according to the presence or absence of *T. gallinae* in oral cavity of the birds. The inoculated birds were ranked based on number of parasites in their oral cavity. The top four were selected to represent susceptible group, and four birds with no parasites in their oral cavity were randomly selected to form the tolerant group. Three birds from uninfected group were selected as a negative control. Selected squabs for RNA sequencing were named as susceptible (S), tolerant (T), and uninfected (N). The remaining squabs were raised for further parasites observation until 8th day of age.

The experimental procedure for rearing and slaughter of the birds was approved (IAS2018-3) by the animal welfare and ethics committee of the Institute of Animal Science, Chinese Academy of Agricultural Sciences.

### Tissue Harvest and RNA Extraction

The selected squabs (S, T, and N) were euthanized by cervical dislocation for the collection of oral mucosa samples, which were immediately stored in liquid nitrogen prior to RNA purification. Total RNA was isolated using Trizol reagent (Invitrogen, Carlsbad, CA, USA) from oral mucosa of susceptible, tolerant and control birds. RNA degradation and contamination were monitored on 1.5% agarose gels. The concentration and integrity of RNA was estimated using the NanoDrop 2000 (Thermo, USA) and Agilent 2100 Bioanalyzer (Agilent Technologies, CA, USA), respectively.

### RNA Sequencing and Assembly

Ribosomal RNA was removed from total RNA prior to sequencing. RNA sequencing was performed using the Illumina Hiseq 2500 platform and 150 bp paired-end reads were generated. The raw reads generated from the 11 libraries were filtered using FastQC (0.11.2). RNA sequencing data in this study was deposited to NCBI Sequence Read Archive (SRA) with accession number PRJNA701112. Clean reads were aligned to the pigeon reference genome (Clv1.0) using HISAT2 with default parameters (14). Reads alignment results were transferred to StringTie v1.3.3b (15) for transcript assembly without using any gene annotations. After the transcriptome assembly, transcripts were merged into the set using the “merge” command in StringTie with default parameters, where pigeon gene transfer format (Clv1.0) was used as annotation.

### LncRNA Identification

In order to explore putative pigeon lncRNAs from the oral mucosa transcriptome, a pipeline was adopted taking cue from previous researches (16, 17). Briefly, we used several strict filters to screen potential lncRNAs from all transcripts. First, transcripts shorter than 200 nt, exons number <1 and without strand information were removed; second, transcripts with class code “=,” “e,” “p,” and “c” were discarded; and third, transcripts with low expression levels (FPKM <0.1) were filtered out, and further searched against CPC, CNCI, CPAT, and Pfam that have the power to distinguish the protein-coding genes from the non-coding genes (18–21). As well as the different types of lncRNAs include lincRNA, intronic lncRNA, anti-sense lncRNA, sense lncRNA were selected using gffcompare. On the basis of the features of the lncRNAs, their localization and abundance were shown using a custom perl script.

### Differential Expression and Enrichment Analysis

Differential expression analysis among three groups was conducted using Deseq2 packages performed in R software. LncRNAs and mRNAs exhibiting an absolute value of fold change > 1.5 and *P*-value < 0.05 were considered as differentially expressed. To investigate the biological function of the DE lncRNAs and mRNAs, pathways analysis by Gene Ontology (GO) was executed. The enriched GO terms including biological process (BP), cellular component (CC), and molecular function (MF) with *P* < 0.05 were considered to be significant. Additionally, Kyoto Encyclopedia of Genes and Genomes (KEGG) analysis was used to test the statistical enrichment of associated genes to predict the possible pathways involved. Both GO and KEGG were carried out in DAVID platform using rock pigeon as background genome.

### Target Gene Prediction and Functional Analysis

The cis role of lncRNAs was defined as those exerting effects on neighboring target genes (22). Coding genes located within 100 Kb upstream and downstream of lncRNAs were checked using in-house Perl scripts. The trans-acting correlation of lncRNA and mRNA was used to identify each other through the expression level (23). The expressed correlations between lncRNAs and coding genes were calculated using the Pearson method. Genes expression level significantly correlated with lncRNAs (absolute *r*-value > 0.9, *P*-value < 0.01) were selected as trans-genes. Cytoscape 3.8.2 were used to plot candidate lncRNA-mRNA network based on the log2 fold change of lncRNA.

### Quantitative Real Time PCR (qRT-PCR) Validation

Total RNA of the same sources and concentrations with library preparation was reverse transcribed into cDNA using PrimeScript RT Reagent Kit (TaKaRa, Japan) following the manufacturer's instruction. Quantitative RT-PCR was performed on ABI QuantStudio 7 Flex Real-time Detection System (Life Technologies Holdings Pte Ltd, USA). Each 10.0 μL PCR mixture contained 5 μL of SYBR Premix Ex Taq™ II, 0.5 μL (10 pM) of each primer, 0.2 μL of ROX Reference Dye II (50 × ), 1.5 μL of cDNA (100 ng), and 2.3 μL of ddH_2_O. Thermo cycling conditions consisted of an initial denaturing at 95°C for 3 min, for 40 cycles of amplification (95°C for 30 s and 60°C for 34 s), followed by thermal denaturing (95°C for 15 s, 60°C for 60 s, and 95°C for 15 s) to generate melting curves to verify amplification specificity. Pigeon β*-Actin* was used as an endogenous control. Relative quantifications of genes were calculated by 2^−ΔΔCT^ method. The primers of randomly selected genes and lncRNAs were designed using Prime3 and NCBI Primer-Blast.

## Results

### Parasite Load

The mean parasite number observed in the oral cavity of the susceptible and tolerant birds at 3 dpi were 3.19 × 10^4^ (± 1.17 × 10^4^, SE) and 0 parasite/mL respectively. *T. gallinae* parasites were not detected in the uninfected group at 3 dpi. Throughout the period of the experiment, umber of parasites counted in susceptible squabs was significantly larger than 0 (Table 1). The number of parasites in the oral cavity of the susceptible squabs rapidly increased to 180.69 × 10^4^ at 7 dpi, although no lesion was observed in the all experimental birds.

**Table 1:** Number of *T. gallinae* in the oral cavity of squabs at different days post infection (dpi)^a^.

**Group^**b**^**	**1 dpi**	**2 dpi**	**3 dpi**	**4 dpi**	**7 dpi**
*N*	0	0	0	0	0
*S*	1.48 ± 0.37	1.66 ± 0.37	3.19 ± 1.17	12.45 ± 9.78	180.69 ± 72.37
*T*	0	0	0	0	0

a *data are presented as mean ± standard error, unit is 10^4^ parasite/ml*.

b *N denotes uninfected birds, S denotes susceptible birds infected with T. gallinae, and T denotes tolerant birds infected with T. gallinae*.

### Identification and Characterization of lncRNAs

**Figure 1:**
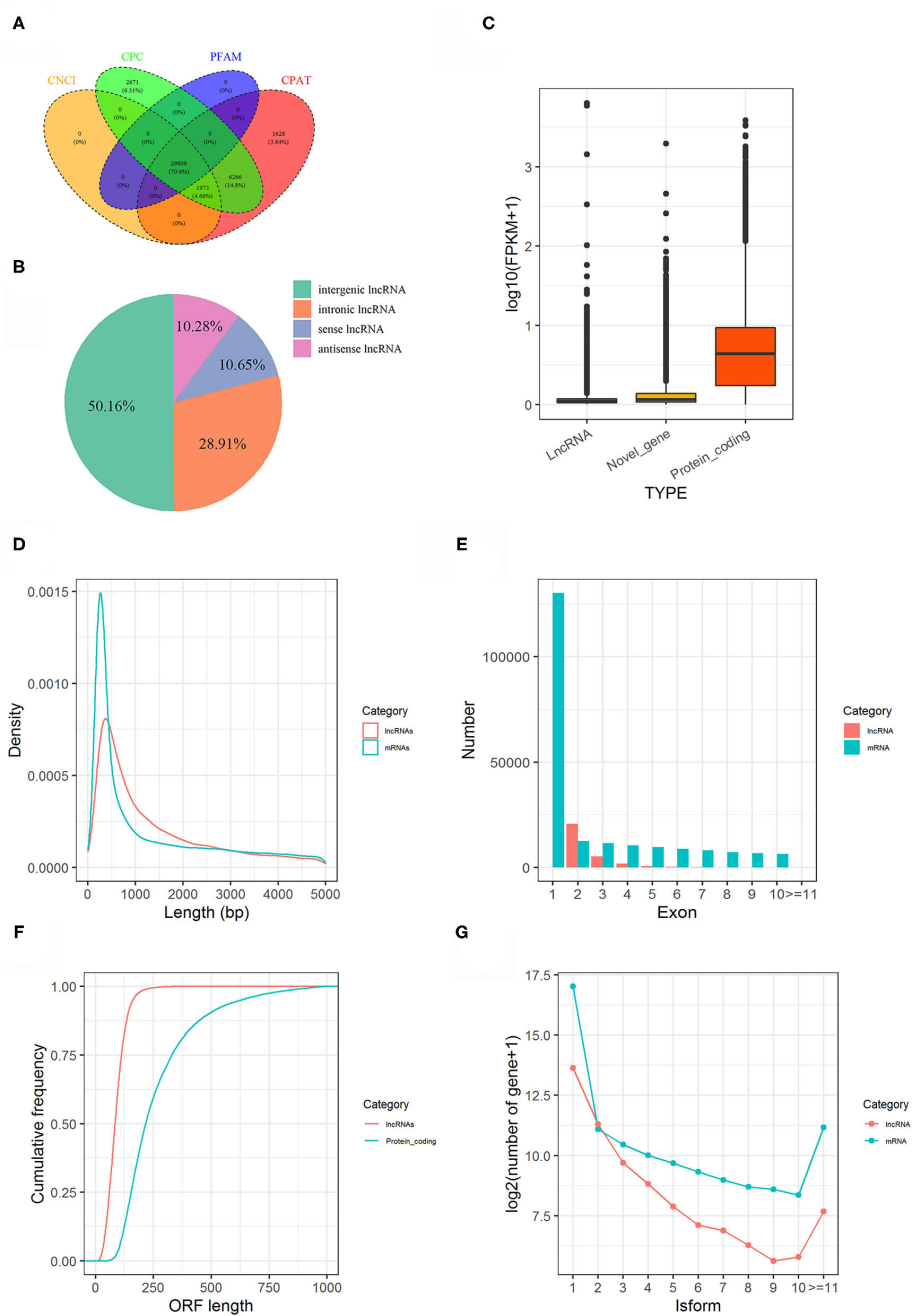
Genome-wide lncRNA prediction and features in pigeon. **(A)** LncRNAs predicted from CNCI, CPC, CPAT, and PFAM. **(B)** Classification of predicted lncRNAs. **(C)** Expression level of lncRNAs, novel genes and protein coding genes. **(D)** Length distribution of lncRNA transcripts compared to mRNA transcripts. **(E)** The number of exons for lncRNA transcripts compared to mRNA transcripts. **(F)** The ORF length of lncRNA transcripts compared to protein-coding genes. **(G)** Distributions of Alternative splicing of pigeon (*Columba livia*) lncRNAs and mRNAs.

In present study, a total of 180.77 gigabytes clean data sets were analyzed after eliminating the low-quality reads. Clean reads were mapped to the pigeon reference genome (Columba livia 1.0) and reads not properly mapped were discarded, resulting in overall mapping rate ranging from 85.65 to 91.45% (Table 2). After prediction with mapped data, a total of 106,204 novel transcripts were obtained, and novel transcripts were further screened for coding potential using CPC, CNCI, Pfam and CPAT packages, resulting in 29,089 putative lncRNA transcripts (Figure 1A). Among these lncRNAs, long intergenic non-coding RNAs accounted for more than half of total amount (14,953), followed by intronic lncRNAs (8,619), sense lncRNAs (3,173), and antisense lncRNAs (3,064), respectively (Figure 1B and Supplementary Table 1).

**Table 2:** The statistics of sequencing data and mapping data.

**Sample ID**	**Clean reads**	**GC content (%)**	**Q30 percent (%)**	**Unique Mapped Reads**	**Unique mapped rate (%)**	**Overall align rate (%)**	**Exon reads rate (%)**	**Intron reads rate (%)**	**Intergenic reads rate (%)**
N1	109,056,738	48.62	94.29	94,413,932	86.57	88.23	54.40	30.00	15.60
N2	109,235,784	48.59	94.34	94,640,180	86.64	88.06	52.70	32.10	15.20
N3	109,881,144	48.58	94.34	94,476,318	85.98	87.22	50.40	33.50	16.10
S1	105,487,548	49.83	94.53	89,605,026	84.94	86.49	56.90	28.10	15.00
S2	111,778,422	47.86	94.17	98,718,710	88.32	89.88	54.80	29.70	15.50
S3	107,605,900	47.3	94.33	96,656,783	89.82	91.45	56.30	28.40	15.30
S4	107,875,526	47.34	93.98	95,869,340	88.87	90.40	56.10	29.60	14.40
T1	105,705,312	47.79	94.15	92,352,807	87.37	89.19	57.70	26.10	16.20
T2	117,058,098	49.05	94.02	98,284,907	83.96	85.65	60.90	24.90	14.20
T3	120,004,466	47.81	94.31	102,552,464	85.46	86.69	54.20	29.80	15.90
T4	120,003,584	48.4	94.53	103,358,505	86.13	87.52	53.20	31.00	15.80

The features of the identified lncRNAs and mRNAs including expression level, length of transcripts, exon number, length of open reading frame (ORF), and their isoforms are shown in Figure 1. Generally, lncRNAs were expressed at low levels than mRNAs (Figure 1C). The length and exon number of lncRNAs were notably shorter and fewer than that of mRNAs (Figures 1D,E). The length of the ORFs of lncRNAs was mostly shorter than 200 AAs, while length of ORFs was mostly ranging from 100 to 750 AAs in mRNAs (Figure 1F). LncRNA transcripts exhibited lower number of isoforms compared to mRNA transcripts (Figure 1G).

### Identification of Differentially Expressed LncRNAs and Genes

**Figure 2:**
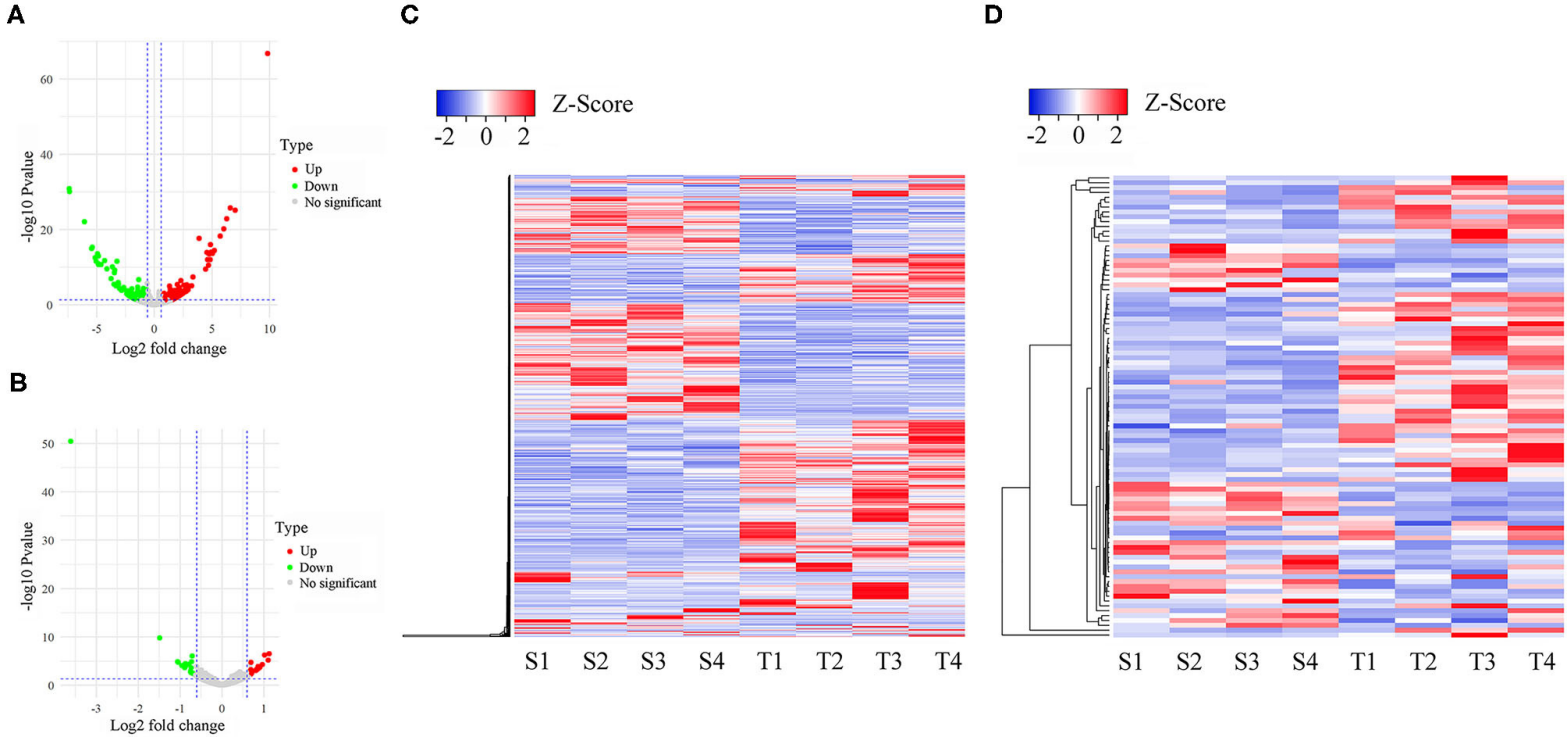
Differentially expressed lncRNAs and mRNAs in susceptible and tolerant pigeons infected with *Trichomoniasis gallinae*. The volcano plot of differentially expressed lncRNAs **(A)** and mRNAs **(B)**. The significantly up- and down-regulated candidates are presented as red or green dots, respectively, the gray dots represent transcripts whose expression levels did not reach statistical significance (fold change > 1.5 and *P* < 0.05). **(C)** Cluster analysis of differentially expressed lncRNAs. **(D)** Cluster analysis of differentially expressed lncRNAs. S1–S4 denotes susceptible birds, and T1–T4 denotes tolerant birds.

PCA analysis showed that the separation of expression among three groups was indistinguishable (Supplementary Figure 2), suggesting that infection did not have a profound effect on the overall oral mucosae transcriptome structure and composition. To identify the differentially expressed lncRNAs (DE-lncRNAs) and genes (DEGs), we performed pairwise comparisons of expression among S, T, and N groups. Heatmaps generated by hierarchical clustering of DE-lncRNAs and DEGs showed a clear separation of the N group from S and T groups (Figure 2 and Supplementary Figures 3, 4). In the S vs. T contrast, a total of 771 lncRNAs were differentially expressed, including 422 up-regulated and 349 down-regulated (Figure 2A and Supplementary Table 2A). Among these DE-lncRNAs, 9 and 11 were uniquely expressed in the S and T groups, respectively. Furthermore, 95 genes were identified to be differentially expressed, of which 61 were upregulated and 34 were downregulated in T vs. S contrast. Only TRMT10C and 2 novel (Columba_livia_newgene_90140 and Columba_livia_newgene_3167) genes were uniquely expressed in S and T group respectively (Supplementary Table 2B). Next, we analyzed DE-lncRNAs and DEGs in the N vs. T contrast, which revealed 828 DE-lncRNAs and 157 DEGs. Among the DE-lncRNAs, 299 of them were upregulated and 529 were downregulated in T, while DEGs contained 76 upregulated and 81 downregulated in T (Supplementary Figure 3 and Supplementary Tables 2C,D). For N vs. S contrast, we identified 890 DE-lncRNAs, with 360 upregulated and 530 downregulated in S, whereas 285 DEGs were identified, including 171 upregulated and 114 downregulated in S (Supplementary Figure 4 and Supplementary Tables 2E,F).

**Figure 3:**
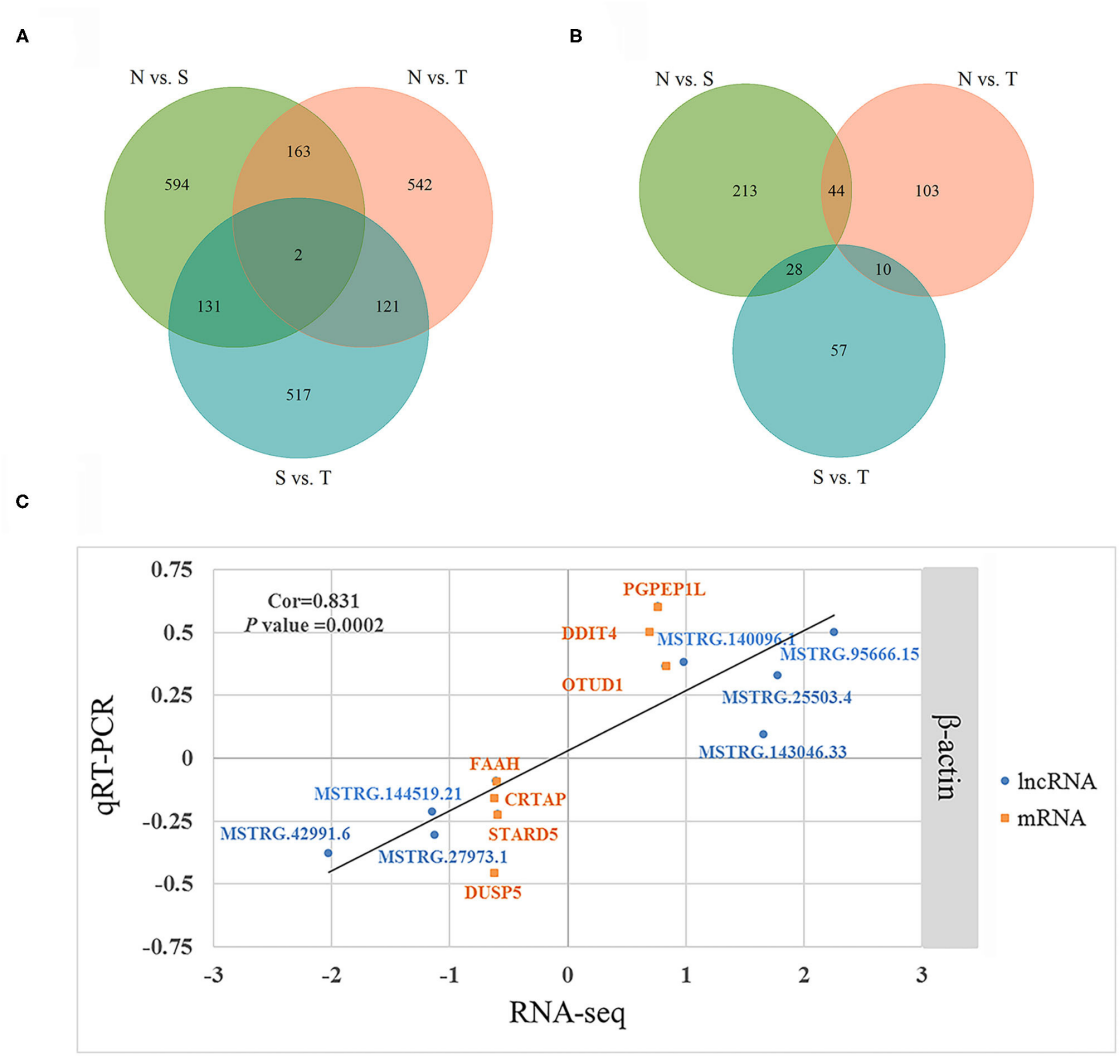
Differentially expressed (DE) lncRNAs and DE genes among birds during *Trichomoniasis gallinae* infection. **(A)** Venn diagram of DE lncRNAs among control (N), susceptible (S), and tolerant (T) birds. **(B)** Venn diagram of DE genes among control (N), susceptible (S), and tolerant (T) birds. **(C)** Correlations of gene expression level of 7 DE genes and 7 DE lncRNAs using RNA-Seq and qPCR. The x- and y-axis represent the relative expression levels measured by RNA-seq and qPCR, respectively. β-actin were used as internal control. The orange and blue dots represent the DE genes and DE lncRNAs, respectively.

We further identified specific intergroup DE-lncRNAs and DEGs that may have profound effect on resistance to *T. gallinae* infection between S and T groups. After dropping DE-lncRNAs and DEGs common to N vs. T and N vs. S contrasts, 517 DE-lncRNAs and 57 DEGs were identified that may play a considerable role in host resistance to *T. gallinae* infection. Among the DEGs, Acetyl-CoA acetyltransferase 2 (ACAT2), acyl-CoA dehydrogenase medium chain (ACADM) which are involved in carbon metabolism. Tolerant birds did not shelter the parasites at 3 dpi, indicating tolerance or resistance to *T. gallinae*. Suppressor of cytokine signaling 3 (SOCS3), immunoglobin superfamily member 21 (IGSF21) and CD101 molecule (CD101), acting as receptors in immune response pathways, also were differentially expressed in the S vs. T contrast exclusively. In the N vs. T contrast, 542 DE-lncRNAs and 103 DEGs were solely differentially expressed, respectively (Figure 3). For the S vs. N contrast, 594 DE-lncRNAs and 213 DEGs were uniquely expressed, respectively (Figure 3). DEGs were significantly enriched in focal adhesion pathway (*P* < 0.05). In addition, two DE-lncRNAs were overlapped among 3 contrasts, however no overlapped DEGs were identified.

To validate the RNA-seq results, 7 DEGs (OTUD1, STARD5, CRTAP, DDIT4, FAAH, PGPEP1L, and DUSP5) and 7 DE-lncRNAs (MSTRG.25503.4, MSTRG.143046.33, MSTRG.42991.6, MSTRG.27973.1, MSTRG.140096.1, MSTRG.144519.21, and MSTRG.95666.15) were randomly selected for qRT-PCR analysis. The primers were listed in Table 3. In Figure 3C, the relative fold change in expression of the selected lncRNAs and mRNAs by qRT-PCR were found to be consistent with the RNA-seq data (*R* = 0.831, *P* = 0.0002). This indicated that our transcript identification and abundance estimation were highly reliable.

**Table 3:** Primers used for quantitative real time PCR.

**Gene name**	**Accession number**	**Primer sequence 5^**′**^-3^**′**^**	**Product size (bp)**	**Tm (^**°**^C)**
*OTUD1*	XM_005500805.2	F: CCATGGGGCAAATGCTGAAC	82	56
		R: TGAACCATGGTGGAAACGGT		
*STARD5*	XM_005513899.2	F: GTTCGCTGGCAACTTGTACC	114	56
		R: ACGGTTTTGTCCCACTTGGT		
*CRTAP*	XM_005502877.2	F: TATTGCGGCAGCTCACACAT	147	57
		R: GCCCTGACAAACAGATTCTCG		
*FAAH*	XM_005501003.3	F: TGCAGCTGTTGTGCTACTGA	139	56
		R: GGTGCCTGGATTCTCTTGCT		
*DDIT4*	XM_021280750.1	F: CTCATCGAGGAGTGCTGACG	116	59
		R: ACAGCCCTACTCCAGCCTAA		
*PGPEP1*	XM_021281574.1	F: TTCGTGTGAAAGTGGAGCGA	149	56
		R: TGGCTTCAGAACTACTTTCATTTCC		
*DUSP5*	XM_021295890.1	F: GCAGAGTGCCAACCACAAAC	51	58
		R: ATTTCAACTGGACCACCCTGG		
MSTRG.25503.4		F: CACAAGTCTATGGGGCCAGA	137	56
		R: TTTGCCAGCTTCCAGTCAAC		
MSTRG.143046.33		F: CGTCTCTTCGCATGCTTAGG	107	58
		R: AGCAGAGTCAGTTCCCAGTG		
MSTRG.42991.6		F: TGTGGTGTGCAGTTTGATGG	83	55
		R: CTGCCAAGGTTTCTGCATT		
MSTRG.27973.1		F: ACCAAGATGCTGAAGGGAGT	143	58
		R: CTCCATCCTCCTGACACTCC		
MSTRG.140096.1		F: CCCTGCCACCCTCTCTTTT	124	59
		R: TTTCCAGGTCCCTTCCATCC		
MSTRG.144519.21		F: AGTGAGGCCTACATCATCCG	125	58
		R: TGATGTGTGTAGGAGGGCTG		
MSTRG.95666.15		F: TGCTCCTATTGCCCTTGACA	82	56
		R: CCTACCTGCTGTAACCCACT		
*β-actin*	XM_005504502.2	F: GAGAAATTGTGCGTGACATCA	152	57
		R: CCTGAACCTCTCATTGCCA		

### Functional Prediction of LncRNAs

**Figure 4:**
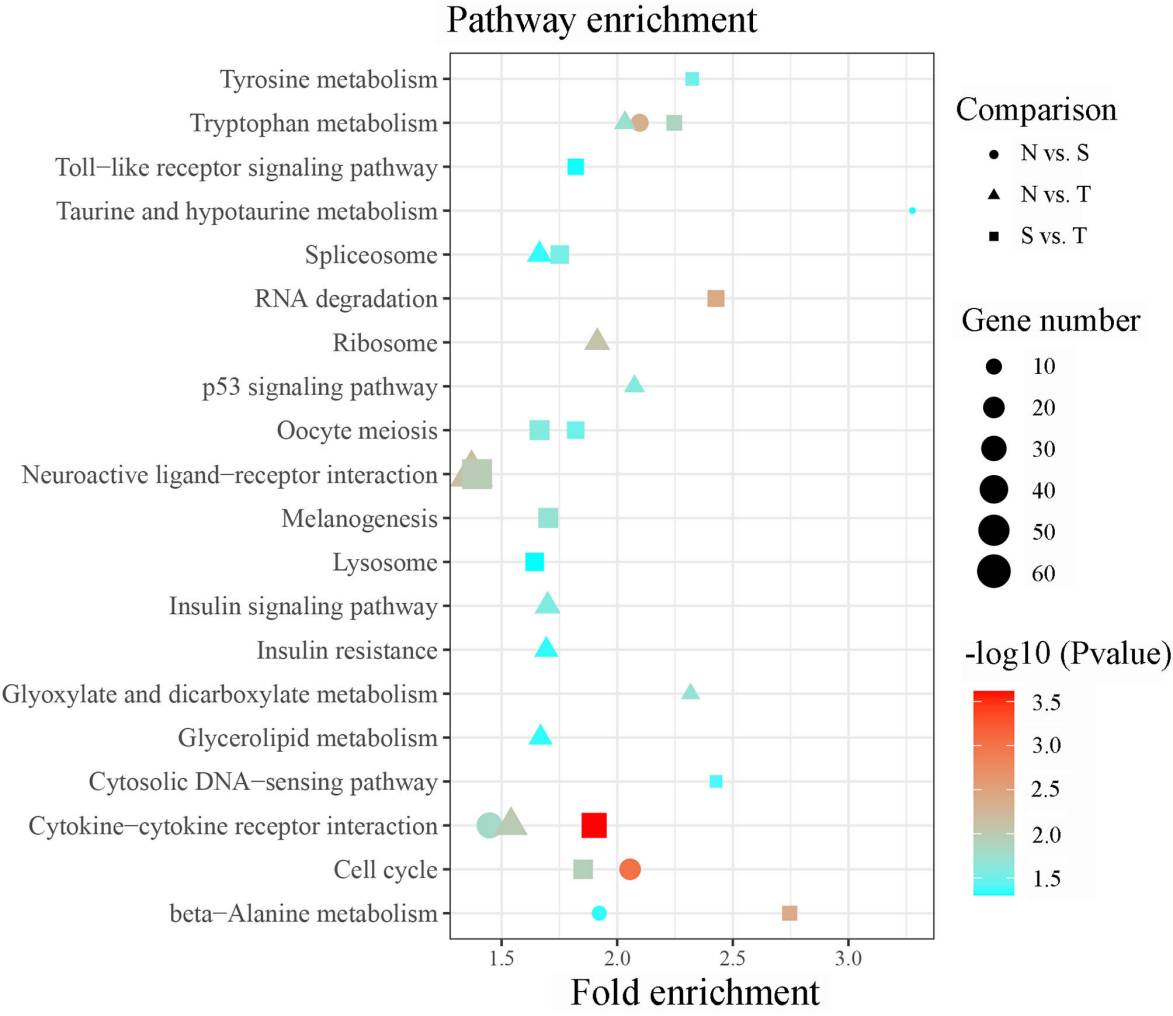
Significant enriched KEGG pathway by cis/trans genes of the differentially expressed lncRNAs within susceptible vs. tolerant birds' contrast.

Briefly, 561 unique DE-lncRNAs targeted 1,635 unique genes *via* cis acting manner whereas 863 unique DE-lncRNAs targeted 6,420 unique genes *via* trans acting manner in N vs. S contrast (Supplementary Tables 3A,B). Similarly, 554 unique DE-lncRNAs targeted 1,609 genes by cis acting manner while 807 unique DE-lncRNAs targeted 5,506 genes by trans acting pattern in N vs. T contrast (Supplementary Tables 3C,D). In the same vein, 1,391 and 5,277 genes were targeted by 459 and 737 unique DE-lncRNAs *via* cis and trans acting manner respectively in S vs. T contrast (Supplementary Tables 3E,F). The GO enrichment analysis indicated that target genes for DE-lncRNAs in N vs. T were significantly associated with nucleotide binding (Molecular function). Conversely, target genes of DE-lncRNAs in S vs. T were enriched in DNA binding (molecular function), and nucleosome and nucleus (cellular components) (Supplementary Table 4). Result of the KEGG pathway enrichment analysis for all the target genes of the DE-lncRNAs in the three contrasts were enriched in several pathways including cytokine-cytokine receptor interaction, cell cycle, RNA degradation, neuroactive ligand-receptor interaction, spliceosome, oocyte meiosis, DNA-sensing pathway, toll-like receptor signaling pathway, ribosome, p53 signaling pathway, and insulin signaling pathway (Figure 4).

### Co-expression Analysis of LncRNA and mRNA

**Figure 5:**
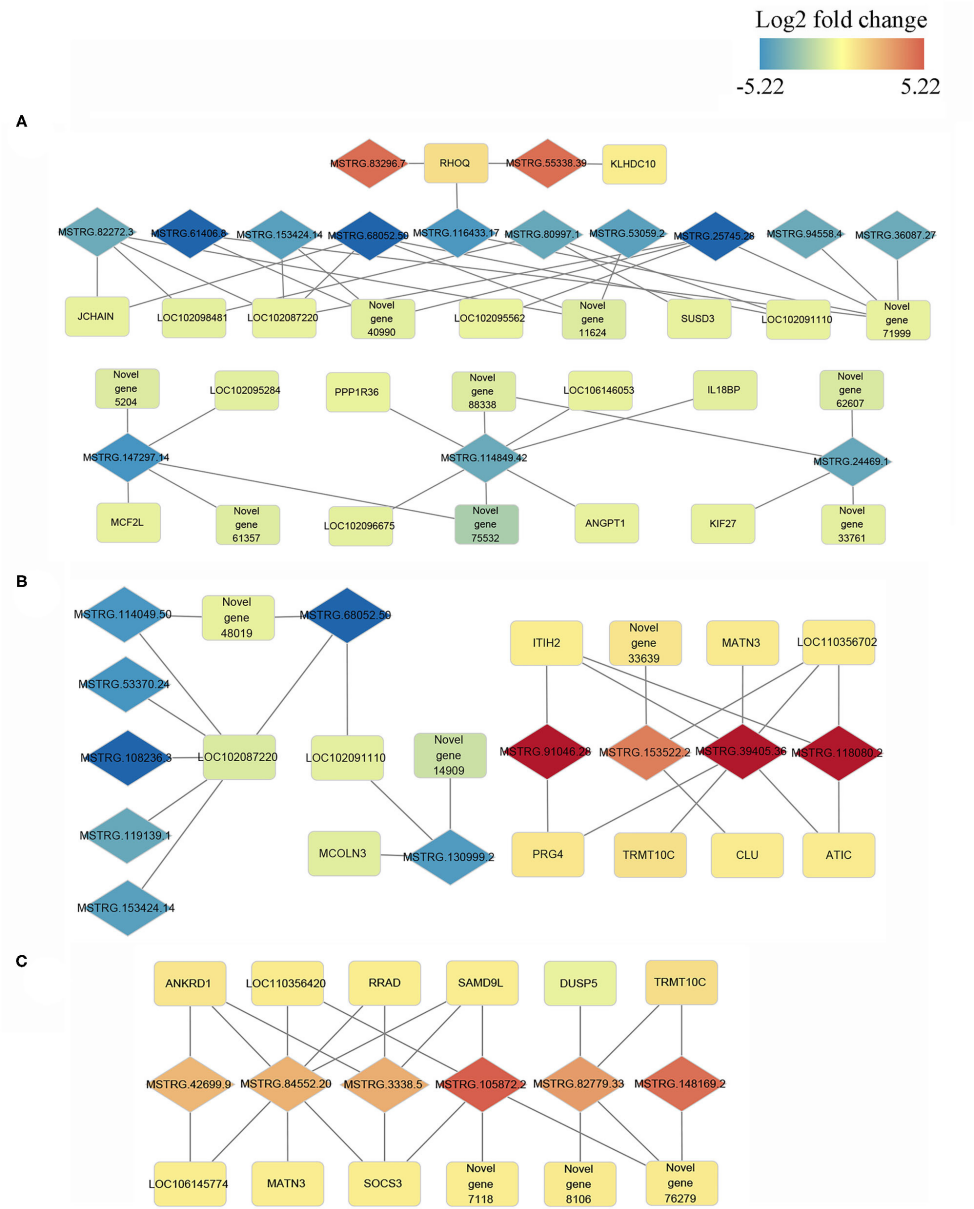
Co-expression network of differentially expressed (DE) lncRNAs with their associated DE genes. Representatives of predicted interaction networks among DE lncRNAs and DE genes within control vs. tolerant birds' contrast **(A)**, within control vs. susceptible birds' contrast **(B)** and susceptible vs. tolerant birds' contrast **(C)**. The gradual color of blue to red diamond and rectangle represent DE lncRNAs and DE genes, respectively. The black solid line denotes the connection of DE lncRNAs and DE genes.

Cis and trans regulatory genes of DE-lncRNAs were further merged with DEGs, resulting in 693, 504, and 388 DE-lncRNA and DEGs co-expression pairs for N vs. S, N vs. T, and S vs. T, respectively (Supplementary Table 5) We consider these DEGs and DE-lncRNAs to be promising for tolerance to the *T. gallinae* infections. We constructed the lncRNA-mRNA network using Cytoscape3.8.2 for the top 10% DEGs-DE-lncRNAs pairs in order to understand the relationship between lncRNAs and *T. gallinae* infection. Result shown that a single lncRNA can potentially regulate multiple protein coding genes, and one protein coding genes could be regulated by more than one lncRNA (Figure 5). For example, MSTRG.114849.42 was linked to PPP1R36, IL18BP, ANGPT1, LOC102096675, LOC106146053, and 2 novel genes. Conversely, TRMT10C was regulated by multiple lncRNAs including MSTRG.39405.36, MSTRG.82779.33, and MSTRG.148169.2 whereas RHOQ was highly correlated with MSTRG.83296.7 and MSTRG.55338.39. In addition, immune-related genes, including ANKRD1, CLU, DUSP5, RRAD, SAMD9L, and SOCS3 contributed to the modulation of host immunity during *T. gallinae* infections through lncRNA-mRNA co-expression network.

## Discussion

*T. gallinae* caused severe lesions by invading the birds' upper digestive tract, in which oral cavity is the earliest damaged organ during infections (24). Infection may likely be influenced by thousands of molecules, including lncRNAs. However, little is known about the non-coding transcriptome in oral mucosa of pigeon in response to Trichomonas infection. In this study, we conducted a preliminary investigation of lncRNA expression profiles in the oral mucosa of white king pigeon squabs to identify the key genes and lncRNAs that are potential regulators of resistance to *T. gallinae* infection. Consequently, the present work provides a catalog of important lncRNAs in pigeon oral mucosa. The number of putative lncRNAs identified in the current study are more than previously reported in chicken, cattle and pig (25). Previous studies in pigeon documented high number of lncRNAs in testis (13) and crop tissues (12). The little differences in the number of lncRNAs among pigeon studies may be attributed to larger sample size and difference in tissue types (23, 26). Significant difference among animals probably resulted from the poor genome assembly of pigeon, which need to be further investigated. LncRNAs were expressed at lower levels than protein coding genes, confirming that lncRNAs are different from mRNAs with respect to their biogenesis, processing, stability, spatial-temporal, and tissue-specific expression patterns (23, 27). The length of lncRNAs are shorter than mRNAs probably due to incomplete genome assembly, resulting in the number of alternative splicing (isoforms) in lncRNAs being less than mRNAs (16). Although RNAs with an ORF <300 nt are often classified as putative non-coding RNAs (28) 4.24% of the expressed mRNAs in the oral mucosa of pigeon squabs in the current study have an ORF lengths shorter than 300 nt. This suggests that mRNAs with short ORF may be more abundant than previously thought. Generally, the lncRNAs identified in the present study exhibited lower expression levels, fewer exons and isoforms, shorter transcripts and ORF lengths than mRNAs, which is consistent with studies in other species (17, 29, 30).

Numerous studies have been conducted recently to unravel genes and/or genetic variants responsible for parasite resistance in humans (31), animals (32), and aquaculture (33). Findings of these investigations linked various immune-related genes and pathways with resistance to parasitic infection. Several genes previously linked to parasite resistance including ACAT2 (34), SOCS3 (35–37), and IGSF21 (38) also showed differential expression pattern between tolerant and susceptible birds. These genes are therefore promising candidates for further validation and practical application. In other studies, IL18BP (39), SOCS6 (40), ANGPTL4 (41), PPP1R36 (42), and NFKBIE (43) were reported to be involved in parasitic resistance in various species. Interestingly, we also identified ANGPTL4 to be up regulated in the tolerant squabs compared to uninfected group and its elevated expression may be triggered by *T. gallinae* infection. In a related investigation, methylation-related genes and heat shock proteins including HEMK1, HSPA8, and HSPBAP1 were reported to be differentially expressed between susceptible and resistant birds, suggesting that epigenetic modulation may contribute to the resistance in parasitic infection in pigeons (44, 45). Here, we also identified another heat shock protein gene (HSPH1) that was differentially expressed in T vs. N and S vs. T comparisons.

Result of the KEGG pathways analysis for DEGs showed that focal adhesion, metabolic pathways and biosynthesis of antibiotics pathways may be associated with *T. gallinae* infections or resistance to it. Genes enriched in focal adhesion such as SHC3, COL6A6, FN1, PARVA, and PTEN are known to participate in cell migration, survival and apoptosis. We suspected that it would likely regulate the interaction between epithelial cells and the extracellular matrix leading to tissue destruction during *T. gallinae* invasion (46). Metabolic pathways and biosynthesis of antibiotics may also be associated with *T. gallinae* infections probably due to the function of genes like UGP2 and OAT that were reported to play a role in nematodes infection (47) and inhibiting *Toxoplasma gondii* (48).

It has been shown in previous studies that lncRNAs act in trans and cis acting manner to regulate the expression of genes (26, 49). The large number of DE-lncRNAs predicted to target multiple protein coding genes in the current study indicates that these lncRNAs might participate in various biological processes in oral cavity. GO functional annotation revealed that the trans and cis target genes of the DE-lncRNAs were enriched in nucleotide binding, DNA binding, nucleosome and nucleus. These terms play a crucial role in regulating cytosine methylation and transcript factor binding (50) and were linked to various biological process including resistance to parasitic infection (51). Furthermore, the target genes were significant enriched in immune-related pathways including toll-like receptor signaling, cytokine-cytokine receptor interaction, neuroactive-ligand receptor interaction and p53 signaling pathways. toll-like receptors are transmembrane pattern recognition receptors that are best known for their roles in innate immunity for the detection of and defense against microbial pathogens (52), and the signaling pathway is conserved from insects to mammals. The role of p53 signaling pathway in *Helicobacter pylori, Chlamydia trachomatis, Shigella flexneri, Plasmodium*, and *Leishmania* infection was widely studied. The activation of p53 induced apoptosis and blocked cell cycle progression of pathogens (53). Additionally, insulin signaling pathway is also enriched in target genes of DE-lncRNAs between susceptible and tolerant squabs. This classic pathway was previously reported to affect the development of multi-cellular parasites. The host insulin pathway regulated nutrient metabolism and controls parasite transmission by blocking its development (54, 55). According to the co-expression network and considering its critical effects on the resistance to *T. gallinae* infections and immune response, five networks with multiple lncRNAs and multiple protein coding genes were retained. In these representative interaction networks, JCHAIN expressed by mucosal and glandular plasma cells regulates polymer formation of immunoglobulin (Ig) A and IgM (56). Mucosal tissues are a primary entry point for infection, and JCHAIN functions in the transport of immunoglobulins to mucosae (57). Novel lncRNAs MSTRG.68052.50 and MSTRG.82272.3 that targeted JCHAIN, may be promising lncRNAs affecting host immune system during *T. gallinae* infection. Further, PPP1R36, IL18BP, and ANGPT1 were predicted to be targets for MSTRG.114849.42, implying the lncRNAs may modulate immune response to *T. gallinae* infection. The expression of MSTRG.153522.2 was highly correlated with ITIH2, MATN3, LOC110356702, PRG4, TRMT10C, and ATIC. These genes were mainly related to the methylation reactions (58), but they were rarely reported in parasitic studies, and therefore need to be further investigated on their role in *T. gallinae* tolerance in squabs. By comparing lncRNAs expression between tolerant and susceptible birds, MSTRG.84452.20 was predicted to target 7 genes that were enriched in p53 signaling (59), NF-κB signaling (60, 61), JAK-STAT pathway (62). All these genes are critical to immune response. Therefore, MSTRG.84452.20 may play a significant role in the resistance to the *T. gallinae*, and could be a key candidate molecular marker for the selection of *T. gallinae* resistance birds.

In conclusion, the expression profiles of mRNA and lncRNA were investigated in the oral mucosa of squabs that showed susceptibility and tolerance to *T. gallinae* infection. Several DE-lncRNAs including MSTRG.82272.3, MSTRG.114849.42, MSTRG.39405.36, MSTRG.3338.5, and MSTRG.105872.2 targeted methylation and immune-related genes, such as JCHAIN, IL18BP, ANGPT1, TMRT10C, SAMD9L, and SOCS3, which are promising lncRNA and genes with potential to confer resistance to *T. gallinae* infection. These molecules need for further validation. Findings in the present study therefore provide novel insights for exploring the molecular markers for identification of tolerance to trichomonas in pigeons.

## Data Availability Statement

The datasets presented in this study can be found in online repositories. The names of the repository/repositories and accession number(s) can be found in the article/Supplementary Material.

## Ethics Statement

The animal study was reviewed and approved by the Institute of Animal Science, CAAS animal care and Use committee (Project number IAS2019-67).

## Author Contributions

JY and AN conducted the experiment, analyzed the RNA-seq data, and wrote the manuscript. AN, YLi, SB, and PG carried out the molecular experiment. YLi, YLiu, PW, LS, and AI contributed to interpretation of results and sample collection. YS contributed to English editing. HM and JC led the project and designed the study. All authors contributed to drafting the work, gave final approval for the version to be published, agreed to be accountable for all aspects of the work in ensuring that questions related to the accuracy or integrity of any part of the work are appropriately investigated and resolved, provided critical feedback, and helped shape the research, analysis, and manuscript.

## Conflict of Interest

The authors declare that the research was conducted in the absence of any commercial or financial relationships that could be construed as a potential conflict of interest.

## Publisher's Note

All claims expressed in this article are solely those of the authors and do not necessarily represent those of their affiliated organizations, or those of the publisher, the editors and the reviewers. Any product that may be evaluated in this article, or claim that may be made by its manufacturer, is not guaranteed or endorsed by the publisher.

## Supplementary Material

Supplementary Figure 1 Chromosomal distribution of predicted lncRNAs. Intronic lncRNA, anti-sense lncRNA, intergenic lncRNA and sense lncRNA were plotted from interior to outside. The outermost layer means chromosome. Each bar denotes the density of lncRNA in the corresponding position. The central photo is white king pigeon.

Supplementary Figure 2 PCA plot of mRNA **(A)** and lncRNA **(B)** expression matrix for (control) N, S (susceptible), and T (tolerant) group.

Supplementary Figure 3 Differentially expressed (DE) lncRNAs and DE mRNAs within control vs. tolerant pigeons. The volcano plot of DE lncRNAs **(A)** and DE mRNAs **(B)**. The significantly up- and down-regulated candidates are presented as red or green dots, respectively, the gray dots represent transcripts whose expression levels did not reach statistical significance (fold change > 1.5 and *P* < 0.05). Cluster analysis of differentially expressed lncRNAs **(C)** and differentially expressed lncRNAs **(D)**. N1–N3 denotes control birds, and T1–T4 denotes tolerant birds.

Supplementary Figure 4 Differentially expressed (DE) lncRNAs and DE mRNAs within control vs. susceptible pigeons. The volcano plot of DE lncRNAs **(A)** and DE mRNAs **(B)**. The significantly up- and down-regulated candidates are presented as red or green dots, respectively, the gray dots represent transcripts whose expression levels did not reach statistical significance (fold change > 1.5 and *P* < 0.05). Cluster analysis of differentially expressed lncRNAs **(C)** and differentially expressed lncRNAs **(D)**. N1–N3 denotes control birds, and S1–S4 denotes susceptible birds.

Supplementary Table 1 Genomic locations and types of predicted lncRNA.

Supplementary Table 2 Significantly differentially expressed (DE) lncRNAs and DE genes among control (N), susceptible (S), and tolerant (T) pigeons. DE-lncRNA **(a)** and DE genes **(b)** within N vs. S; DE-lncRNA **(c)** and DE genes **(d)** within N vs. T; DE-lncRNA **(e)** and DE genes **(f)** within S vs. T.

Supplementary Table 3 The cis/trans target genes of differential expressed (DE) lncRNA among control (N), susceptible (S), and tolerant (T) pigeons. The protein-coding genes within 100 k upstream and downstream of DE-lncRNA with in N vs. S birds **(a)**, with in N vs. T birds **(c)** and with in S vs. T birds **(e)**. The predicted target genes of DE-lncRNA by trans in N vs. S birds **(b)**, N vs. T birds **(d)** and S vs. T birds **(f)**.

Supplementary Table 4 Significant gene ontology (GO) terms for cis/trans target genes of DE-lncRNAs.

Supplementary Table 5 Co-expressed pairs of differentially expressed (DE) lncRNA and DE genes within control vs. susceptible birds **(a)**, control vs. tolerant birds **(b)**, and susceptible vs. tolerant birds **(c)**.
