# Supplementary figures and images for: Transcriptome Analysis Revealed Potential Mechanisms of Resistance to Trichomoniasis gallinae Infection in Pigeon (Columba livia)
Source: Front Vet Sci. 2021 Sep 14;8:672270. doi: 10.3389/fvets.2021.672270 (PMC8477972; doi:10.3389/fvets.2021.672270)

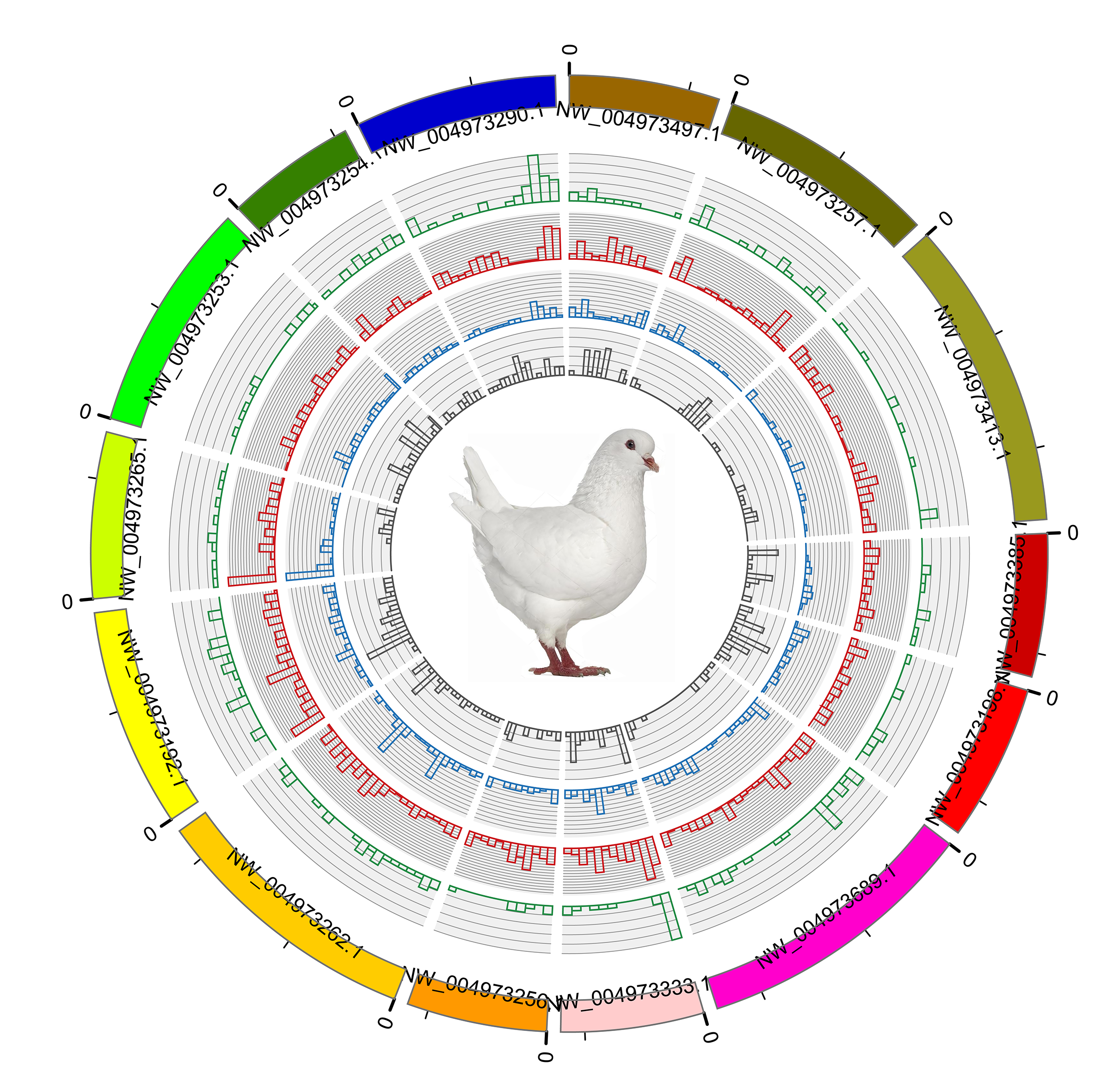

Supplement: Supplementary Figure 1 — Chromosomal distribution of predicted lncRNAs. Intronic lncRNA, anti-sense lncRNA, intergenic lncRNA and sense lncRNA were plotted from interior to outside. The outermost layer means chromosome. Each bar denotes the density of lncRNA in the corresponding position. The central photo is white king pigeon. [file Image_1.TIF]

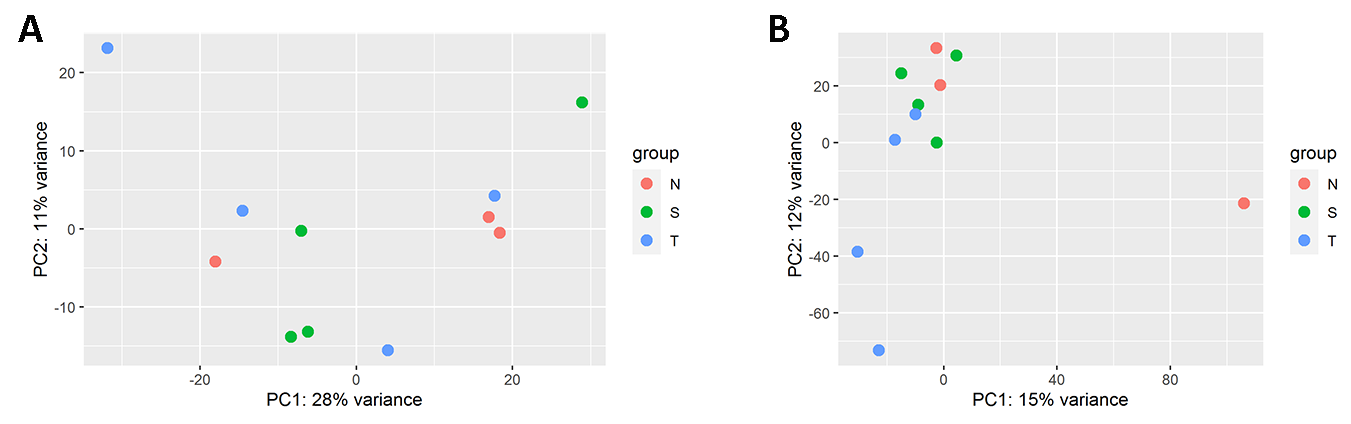

Supplement: Supplementary Figure 2 — PCA plot of mRNA (A) and lncRNA (B) expression matrix for (control) N, S (susceptible), and T (tolerant) group. [file Image_2.tif]

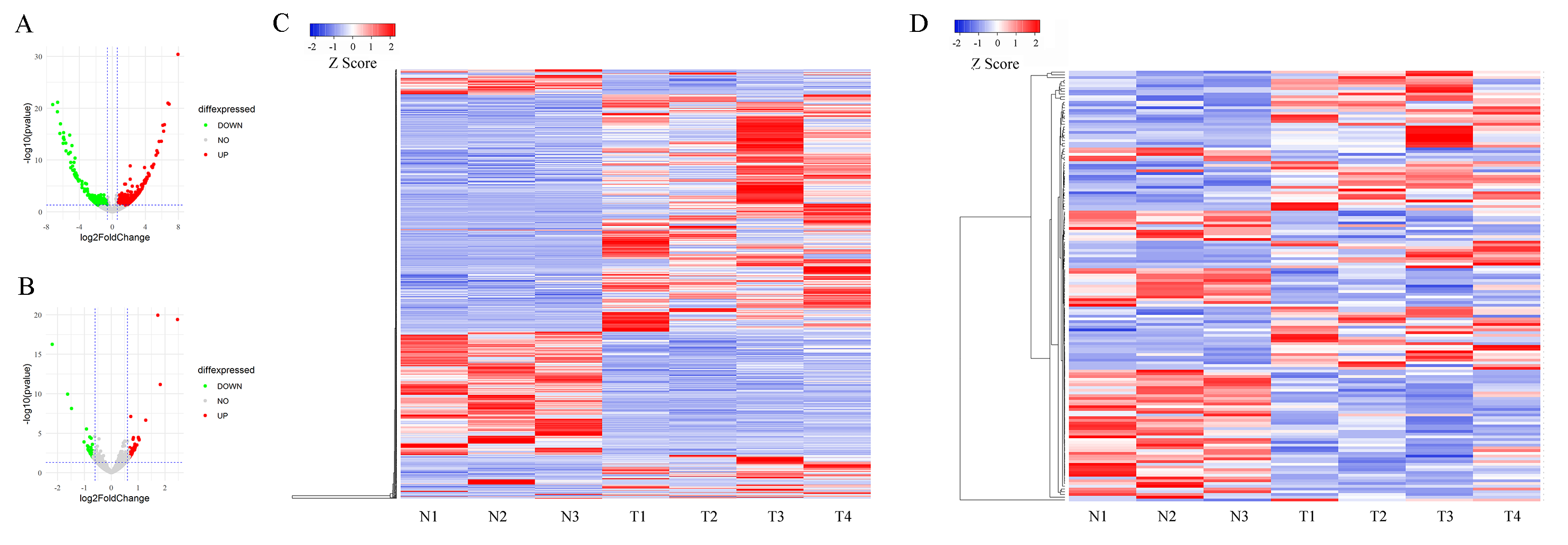

Supplement: Supplementary Figure 3 — Differentially expressed (DE) lncRNAs and DE mRNAs within control vs. tolerant pigeons. The volcano plot of DE lncRNAs (A) and DE mRNAs (B). The significantly up- and down-regulated candidates are presented as red or green dots, respectively, the gray dots represent transcripts whose expression levels did not reach statistical significance (fold change > 1.5 and P < 0.05). Cluster analysis of differentially expressed lncRNAs (C) and differentially expressed lncRNAs (D). N1–N3 denotes control birds, and T1–T4 denotes tolerant birds. [file Image_3.TIF]

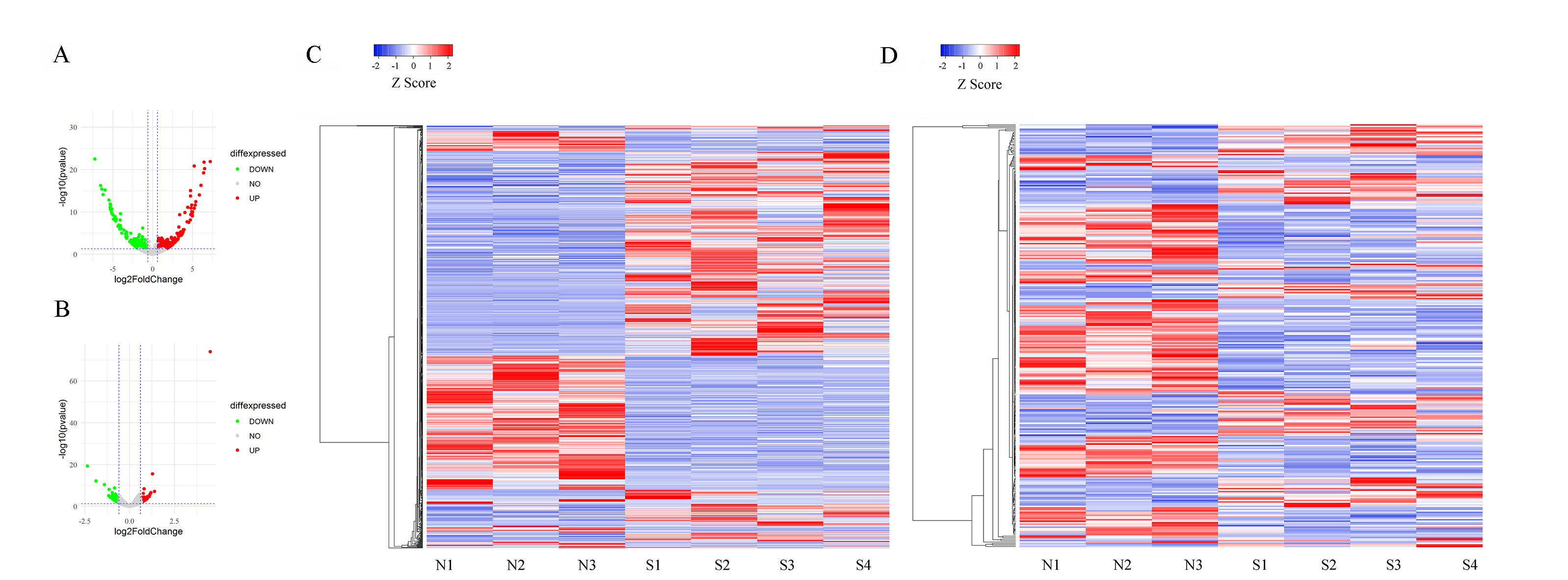

Supplement: Supplementary Figure 4 — Differentially expressed (DE) lncRNAs and DE mRNAs within control vs. susceptible pigeons. The volcano plot of DE lncRNAs (A) and DE mRNAs (B). The significantly up- and down-regulated candidates are presented as red or green dots, respectively, the gray dots represent transcripts whose expression levels did not reach statistical significance (fold change > 1.5 and P < 0.05). Cluster analysis of differentially expressed lncRNAs (C) and differentially expressed lncRNAs (D). N1–N3 denotes control birds, and S1–S4 denotes susceptible birds. [file Image_4.TIF]
